# Virtual reality based attentional bias modification training for patients with obsessive-compulsive disorder: a feasibility study

**DOI:** 10.3389/fpsyt.2026.1688089

**Published:** 2026-06-18

**Authors:** Zijun Yan, Xu Ma, Pengchong Wang, Zhanjiang Li

**Affiliations:** Beijing Key Laboratory of Mental Disorders, National Clinical Research Center for Mental Disorders & National Center for Mental Disorders, Beijing Anding Hospital, Capital Medical University, Beijing, China

**Keywords:** obsessive compulsive disorder, attentional bias modification training, virtual reality, eye tracking, feasibility study

## Abstract

**Background:**

Attentional bias modification (ABM) training is employed to modulate attentional bias and alleviate clinical symptoms in obsessive-compulsive disorder (OCD) patients. This study aimed to develop a novel, ecologically valid ABM paradigm based on virtual reality (VR). The intent was to enhance the efficacy of traditional computerized ABM and to preliminarily examine its feasibility in patients with contamination-based obsessive-compulsive disorder (OCD).

**Method:**

15 patients with OCD who were currently receiving pharmacotherapy were recruited and allocated to either an ABM group, which received VR-ABM training twice weekly for four weeks (eight sessions in total), or a TAU group, which continued their routine outpatient treatment, including pharmacotherapy, psychotherapy, and physical therapy.

**Result:**

5 patients in ABM group and 5 patients in TAU group completed the 4-week follow-up. VR-ABM paradigm effectively elicited target symptoms, with virtual scenarios inducing moderate levels of obsessive-compulsive symptoms and anxiety. Notably, all participants in the VR-ABM group reported the program to be appropriately challenging, effectively eliciting obsessive-compulsive symptoms and improving subjective concentration.

**Conclusion:**

These preliminary findings support the feasibility of the novel VR-ABM training in OCD and warrant further investigation in larger, controlled trials to rigorously evaluate its therapeutic efficacy.

## 1 Introduction

Obsessive-compulsive disorder (OCD) is a common mental disorder characterized by persistent, intrusive, unnecessary obsession and repeated compulsion (1). Residual symptoms remain in 40% -60% of patients after medication or psychotherapy (2). Therefore, there is a pressing need to explore effective adjunctive interventions targeting dysfunctional cognitive processing, such as attentional bias (3), thereby optimizing the effectiveness of standard clinical approaches.

Converging evidence indicates that individuals with OCD exhibit attentional bias towards threaten or disorder-relevant stimuli, suggesting a potential association with obsessive-compulsive symptoms (OCS) (3–6). It has been proposed that attentional control, a higher-order regulatory mechanism, may modulates abnormal attentional bias. Importantly, attentional control possesses considerable plasticity, implying its susceptibility to modification and adaptation (7). Attentional bias modification (ABM) has been employed in OCD to regulate attentional bias (8). According to attentional control theory (9), aberrant attentional bias in OCD may reflect insufficient top-down cognitive control, which may contribute to difficulty disengaging attention from symptom-relevant stimuli. ABM may help alleviate this difficulty by repeatedly training goal-directed attention toward non-threatening stimuli, thereby enhancing attentional control processes and potentially modulating activity in the dorsal attention network and executive control-related frontoparietal systems (10). Previous studies have provided preliminary evidence that ABM may alter attention-related processing in OCS, with multisession online ABM reducing attentional bias and being accompanied by reductions in self-reported OCS (8). However, the clinical efficacy of ABM in OCD has not been established consistently. Hendges found ABM to be feasible and well tolerated, but it did not significantly outperform sham training in changing attentional bias or reducing OCS (11). Further mechanistic studies and better-designed intervention trials are needed to clarify the relationships among attentional bias, ABM, and symptom change in OCD.

Traditional ABM paradigms for OCD are primarily conducted in laboratory settings, utilizing images or words as stimuli. In such controlled environments, participants may exhibit a diminished perception of threat relevance, thereby limiting the allocation of attentional resources to high-threat materials (12–14). Furthermore, the inherent limitations of laboratory contexts and the stimuli employed often compromise ecological validity, thereby hindering the generalization of training effects to real-world scenarios (15, 16).

Compared with traditional computer-based ABM, VR may offer more realistic and immersive symptom-related contexts, thereby enhancing ecological validity and symptom provocation (17). Previous studies have shown that VR can effectively provoke emotional and anxiety responses, and in OCD, VR-based environments have been validated as tools for provoking symptom-relevant anxiety and compulsive urges, particularly in contamination-related symptoms (18). In addition, immersive VR may enhance presence, engagement, and attentional involvement, which could be advantageous given the repetitive and potentially monotonous nature of conventional ABM tasks (12). In this context, VR-based ABM (VR-ABM) may offer a more engaging and controlled training environment for individuals with OCD, potentially improving both the ecological validity and the therapeutic relevance of ABM interventions (19, 20). VR-ABM has been employed to modulate attentional biases towards various stimuli, including threaten face (12, 16, 20), food (21), smoking cues (13), pain-related stimuli (22), and to alleviate self-reported anxiety associated with stress (23). Although the effects on attentional bias were not entirely consistent, participants showed improvements in behavioral responses and anxiety symptoms, together with high engagement and motivation, underscoring the potential of VR-ABM as a promising intervention approach. There have been several case studies or proof-of-concept studies have examined the effect of VR based exposure therapy in OCD, a notable gap remains in the literature, as no published clinical research have specifically examined the efficacy of VR-ABM for OCD, thereby highlighting the novelty and potential contribution of the present study.

Capitalizing on the potential of VR as an emerging digital therapeutic modality for mental health, this study aimed to innovate in both technically and programmatically by establishing a novel VR-based ABM training program. First, in contrast to the previous VR-ABM approaches employing dot-probe tasks, we utilized visual search task within immersive, symptom-relevant environments under conditions designed to elicit symptom provocation. Secondly, considering the single-session VR-ABM interventions might hinder training efficacy and the transfer of learning to real-world settings, we developed a structured 4-week short-term intervention program to enhance both the effectiveness and ecological validity of the training.

Our program utilized standardized VR training scenes, with scene types covering public restroom, hospital, and garbage station. Moving beyond traditional dot-probe tasks, we implemented a visual search task under the provocation of symptoms in ecologically relevant scenes. Attentional allocation patterns were trained by manipulating the proportion of detection targets presented on symptom-laden stimuli (e.g., stains, bloodstains, vomit, urine, infectious disease-related media) compared to clean areas. A brief (500ms) presentation time was selected to specifically target the attentional disengagement difficulties characteristic of OCD. Furthermore, recognizing the limitations of single-session VR-ABM protocols, and informed by previous ABM studies demonstrating efficacy with twice-weekly training over a four-week period, we adopted a same intervention schedule.

Previous studies have yielded inconsistent results, according to the theory of attentional control (9) and the Cognitive-Motivational Framework (24), cognitive function, especially working memory, can affect attentional bias and ABM training outcomes. Studies have shown that low working memory load can promote the effect of ABM training, while high working memory load will impair its effectiveness (25–27). It was also found that increasing working memory load may reduce the effect of ABM in individuals with low working memory capacity, but not in individuals with high capacity (28). Consequently, we not only optimized the training paradigm but also accounted for the influence of cognitive function, refining the evaluation tool for measuring attentional bias. Using eye-tracking technology, we measured attentional bias by combining a visuospatial working memory task with a free-viewing paradigm.

Presenting a novel ABM training paradigm for individuals with OCD, this study sought to evaluate the feasibility of employing VR technology within ABM interventions, with the goal of informing the development of more effective clinical strategies.

## 2 Method

### 2.1 Participants

Patients with OCD were recruited from the outpatient department of Beijing Anding Hospital, all of whom were receiving stable selective serotonin reuptake inhibitors (SSRIs) treatment regimens. Given that different psychotropic medications may affect symptom presentation and cognitive processing, and that treatment regimens in patients with OCD show a certain degree of heterogeneity, we limited inclusion to patients receiving SSRI pharmacotherapy to control pharmacological heterogeneity and to evaluate VR-ABM in a more clinically homogeneous adjunct-treatment context. The inclusion criteria for patients with OCD were: (1) age 18–60 years old, junior high school education or above, to ensure that participants could adequately understand and complete the study-related tasks; (2) meet the diagnostic criteria for OCD in DSM-IV; (3) Yale-Brown Obsessive-Compulsive Scale (Y-BOCS) total score ≥7; (4) have compulsive washing symptoms; (5) right-handed, normal vision or corrected vision, no color blindness and weak color; (6) voluntary participation in the study with informed consent. Patients were excluded from participating in the study if they met the diagnosis of schizophrenia, mood disorders, or other mental disorders, had recently received physical therapy such as modified electroconvulsive therapy or neuromodulation, had a history of organic brain diseases and major physical diseases, had evidence of drug dependence and psychoactive substance use, or were pregnant, breastfeeding, claustrophobia and other subjects who cannot perform VR.

As this was an exploratory pilot feasibility study, no formal *a priori* power calculation was conducted to test treatment efficacy. The primary aim was to evaluate the feasibility, acceptability, tolerability, and preliminary applicability of the VR-ABM program, rather than to provide a confirmatory test of clinical effectiveness. Therefore, the sample size was determined pragmatically based on the availability of eligible patients, the feasibility of recruitment within the study period, and the resources required to deliver repeated VR-ABM sessions. A total of 15 participants were enrolled. Participants were initially randomized to either an ABM group (n=8), which received VR-ABM training twice a week for 4 weeks, or a TAU group (n=7), which continued their routine outpatient treatment, including pharmacotherapy, psychotherapy, and physical therapy. However, before treatment initiation, final group allocation was adjusted according to patient preference, resulting in 6 participants in the ABM group and 9 in the TAU group. All participants underwent assessments of outcome measures, namely assessments for OCS (Y-BOCS, OCI-R), anxiety (BAI), depression (BDI), and attentional bias, at baseline (0 weeks) and at the four-week follow-up. One participant in the ABM group dropped out after the first VR-ABM training session because the participant perceived the treatment effect to be limited and did not wish to continue. Four participants in the TAU group did not complete the 4-week follow-up assessment because they were unable to return to the hospital. More detailed reasons for their failure to attend the follow-up assessment were not available. Finally, there were 5 patients in the ABM group and 5 patients in the TAU group who completed the 4-week follow-up. Patient’s details are shown in **Table 1**.

**Table 1:** Patient condition.

ID	Group	Gender	Age	Education	Medicine
1	ABM	female	32	Undergraduate	Sertraline
2	ABM	male	31	Junior college	Sertraline
3	ABM	male	36	Master’s degree	Fluvoxamine, Aripiprazole
4	ABM	male	44	Junior college	Sertraline
5	ABM	male	29	Undergraduate	Fluvoxamine
6	TAU	female	26	Master’s degree	Sertraline
7	TAU	male	26	Undergraduate	Fluvoxamine
8	TAU	male	40	Master’s degree	Fluvoxamine
9	TAU	female	43	Junior college	Sertraline
10	TAU	female	20	Undergraduate	Sertraline

### 2.2 Measures

Mini International Neuropsychiatric Interview (M.I.N.I.) used to screen out other mental disorders (29). The Yale-Brown Obsessive-Compulsive Scale (Y-BOCS) was used to measure the severity of OCS (30). The scale consists of 10 items across two dimensions, obsessive thoughts and compulsive behaviors, with higher scores indicating greater symptom severity. The Chinese version demonstrated good inter-rater reliability (r = 0.75), test-retest reliability (r = 0.91), and satisfactory structural validity. Obsessive-compulsive Inventory-Revised (OCI-R) consists of 18 items and assesses OCS from the dimensions of washing, obsessing, hoarding, ordering, checking, and neutralizing (31). The Chinese version of the OCI-R has demonstrated good internal consistency (Cronbach’s α = 0.84) and excellent test-retest reliability (r = 0.96). Beck Anxiety Inventory (BAI) (32) and Beck Depression Inventory (BDI) (33) were used to assess the severity of anxiety and depression, respectively. Both are 21-item scales rated on a 4-point scale from 0 to 3, with higher scores indicating greater symptom severity. The Chinese version of the BAI demonstrated good internal consistency (Cronbach’s α = 0.95), while the Chinese version of the BDI showed good internal consistency (Cronbach’s α = 0.94) and acceptable test-retest reliability (r = 0.55).

At the same time, we prepared our own ABM feedback form to evaluate the patients’ reaction to ABM under VR. The Subjective Units of Distress Scale (SUDs)-based measure was designed to capture subjective feedback on several aspects, including the degree of symptom and emotion induction and the level of subjective focus. Patients rated these items on a scale from 0 to 10. In addition, the form included questions about patients’ thoughts and performance during the training process, as well as comments and suggestions for improving the procedure.

### 2.3 VR-ABM

#### 2.3.1 VR scene construction

**Figure 1:**
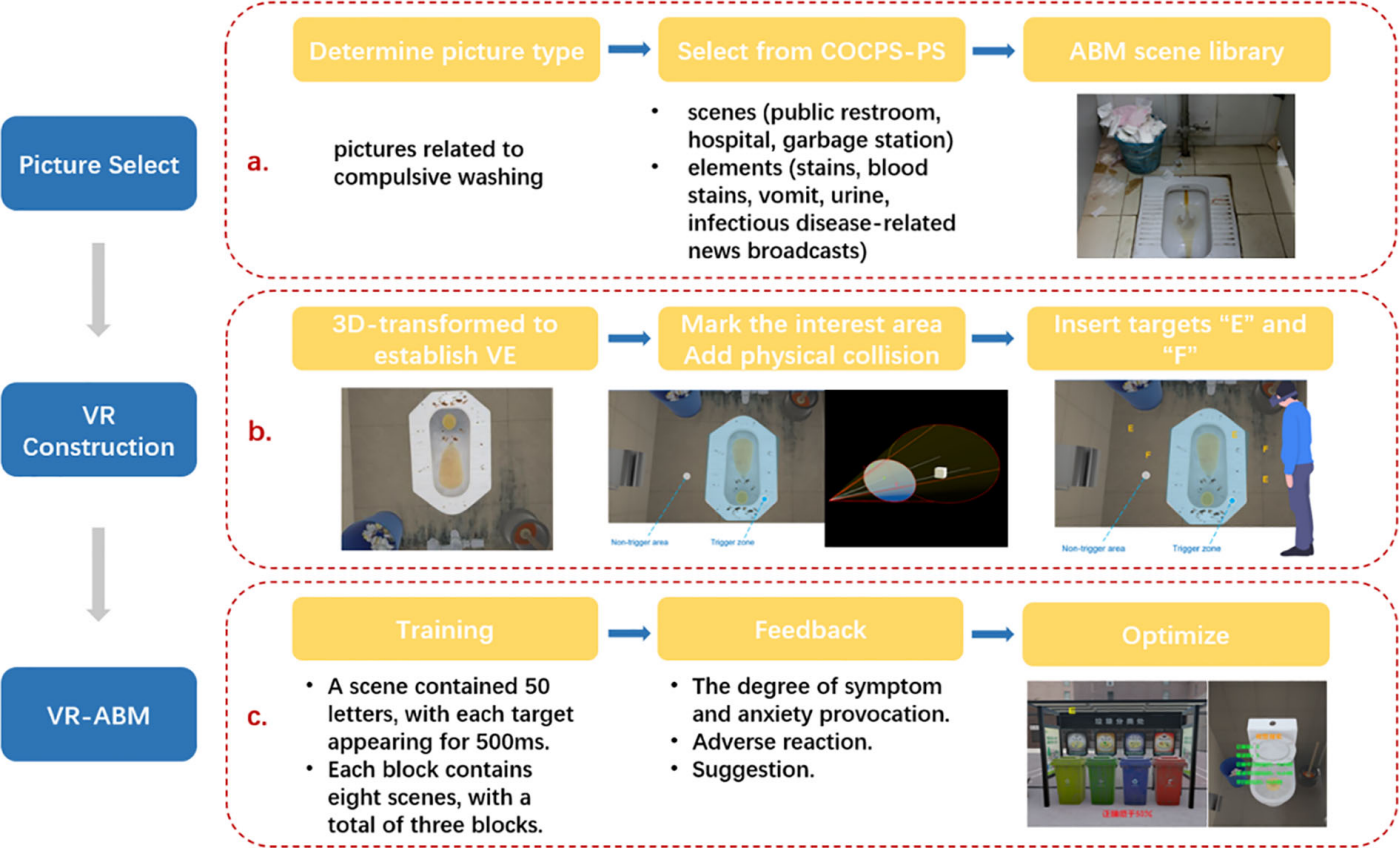
The specific process of VR-ABM program construction. **(a)** is the stage of picture selection. Select the pictures related to compulsive washing from COCPS-PS to construct the scene library for ABM training. **(b)** represents the stage of building the VE. First, 3D-transformed the picture, mark the area of interest in the VE. A physical collision mechanism is added for collision detection. Then the target letters E and F are inserted, and the target is avoided from the region of interest by the sample elimination algorithm. **(c)** is the construction phase of the VR-ABM. According to the task design, complete the construction of the training program.

Based on standardized Chinese obsessive-compulsive symptom provocation picture system (COCSP-PS) (34), the pictures related to contamination were selected and three dimensions (3D)-transformed to establish a virtual environment (VE). In the VE, the scenes were rendered and baked, and a head-tracked perspective was used. The virtual camera was locked to the head-mounted display to ensure that the scene moved synchronously with the user’s head movements. In each scene, the areas corresponding to each triggering element were defined, a physical collision mechanism was added, and physical properties were assigned to objects in the areas of interest to enable collision detection. Targets “E” and “F” were inserted into the scenes, and their presentation was randomized. In addition, an area-of-interest sample removal algorithm was implemented to prevent targets from appearing within the predefined areas of interest, thereby correcting users’ attentional bias in the VE. Before the formal study began, several refinements were made to the product based on feedback from experimenters during the user-experience phase. These adjustments mainly involved the types of scenarios, the duration of the protocol, the type of targets, and their presentation duration. After the study was initiated, all participants experienced the same standardized scenarios and procedures, and no further adjustments were made to the system. In the future, we will continue to optimize and refine the intervention protocol and procedures based on the findings from the preliminary proof-of-concept and pilot studies (**Figure 1**).

#### 2.3.2 Scene assignment

There are 12 VR-ABM training scenes. Each training session consists of 3 blocks, with each block containing 8 scenes. **Tables 2** and **3** show the scene contents and distribution.

**Table 2:** Scene contents.

Number	Contents
Scene 1	public restroom door handle with stains
Scene 2	toilet with blood stains
Scene 3	garbage station
Scene 4	ground with water stains
Scene 5	a hospital broadcasting HIV-related news
Scene 6	toilet with vomit
Scene 7	door of public restroom
Scene 8	a hospital broadcasting news about hepatitis
Scene 9	toilet with urine stains
Scene 10	toilet with poop
Scene 11	stained floor
Scene 12	a hospital broadcasting news related to coronavirus

**Table 3:** VR scene allocation.

Trial	Block 1	Block 2	Block 3
1	scene 1	scene 8	scene 4
2	scene 12	scene 9	scene 2
3	scene 3	scene 7	scene 5
4	scene 4	scene 2	scene 9
5	scene 6	scene 3	scene 11
6	scene 5	scene 6	scene 7
7	scene 11	scene 12	scene 10
8	scene 10	scene 1	scene 8

#### 2.3.3 Training setting and process of VR-ABM

Patients should complete 8 sessions of training twice a week. In the first session, we explained the principles of attentional bias and the significance of ABM training, realized the conditions of the patients, and assessed the severity of symptoms and attentional bias at baseline. At the same time, let the patients get familiar with the operation of VR, and practice until the accuracy exceeds 50%. During formal ABM training, scenes that provoke OCS were presented on the screen as the background for the task. Target E and F appeared randomly on the screen. A scene contained 50 letters, with each target appearing for 500ms. If target E appears, click on the left handle panel, and if target F appears, click on the right handle panel. The screen showed whether patients clicked correctly or not and recorded the subjects’ reaction time and accuracy. Each block contains eight scenes, with a total of three blocks. Training for each session takes about half an hour. At the end of each training, we sought feedback from the patients, including how they felt and whether they had any side effects.

**Figure 2:**
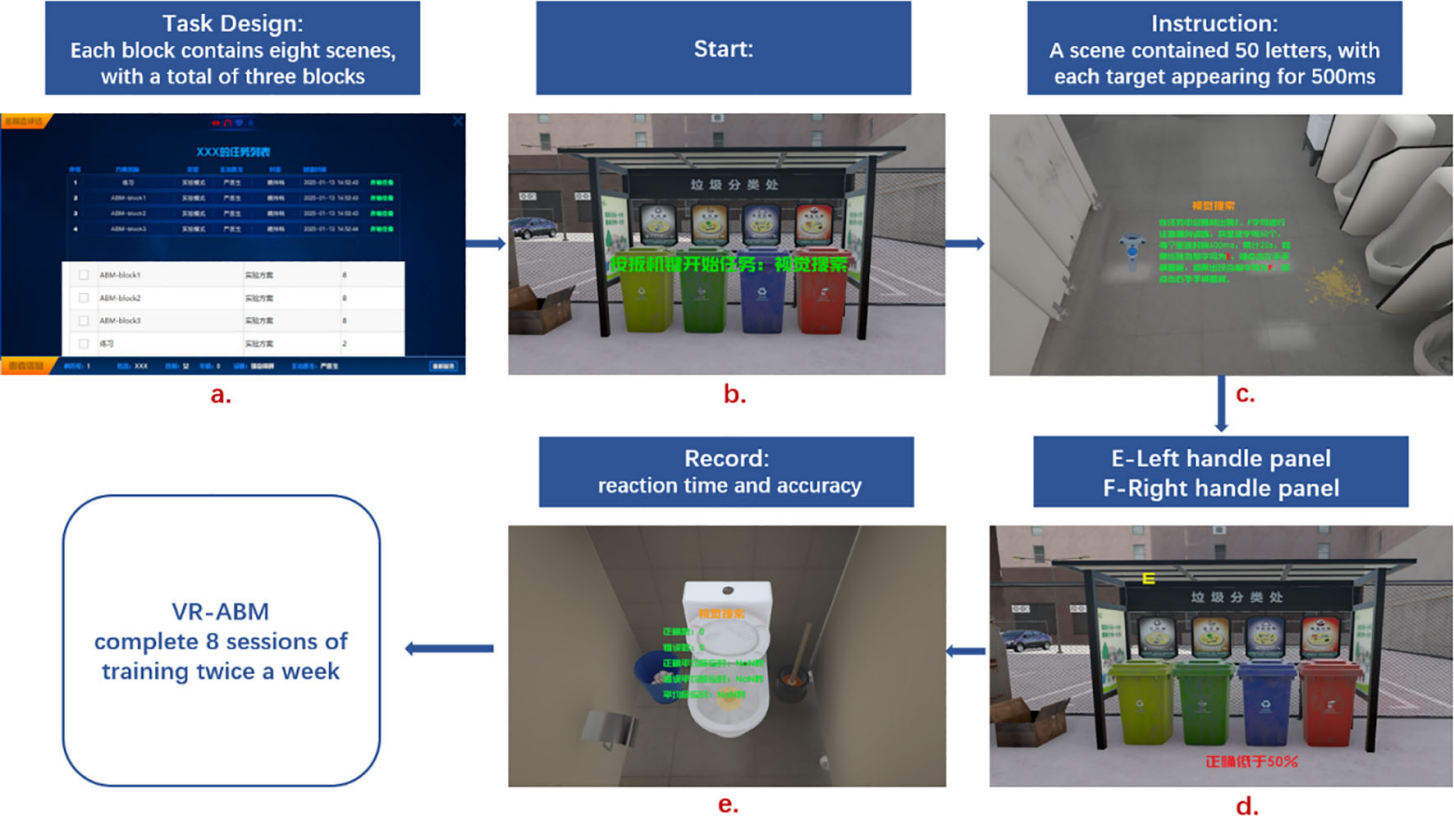
VR-ABM training flow chart. **(a)** is the task allocation interface. During the practice phase, 2 scenes are assigned. The formal training is divided into 3 blocks, with 8 scenes assigned to each block. **(b)** shows the task start screen. Press the trigger button, participants will start the visual search task. **(c)** is the task rule introduction interface. The screen will randomly appear the letters E and F. There are a total of 50 presentations, each lasting 500ms for a total of 25s. If the letter E appears, click the left-hand handle panel. If the letter F appears, click on the right-hand handle panel. **(d)** is for the task interface. After clicking the handle panel, screen will feedback whether it is right or wrong. **(e)** is the feedback interface after each scene ends, including the number of correct responses, the number of errors, the average response time of correct responses, the average response time of errors, and the average response time.

After the 1st, 4th and 8th training sessions, the degree of subjective attention concentration, task difficulty and symptom provocation were also assessed. After the last training session, patients were asked to discuss and seek feedback on the overall 8-session training process, assess the changes in attentional bias and symptoms, and check whether there were adverse events and timely intervention (**Table 4**; **Figure 2**).

**Table 4:** The contents of each session of the VR-ABM program.

Session	Content		Note		Outcome Measures
Session 1	Explain the principle of attentional bias and the significance of ABM & Practice When the accuracy is greater than 50%, formal training begins.	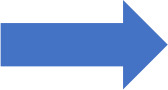	Assess baseline symptom severity and attentional bias	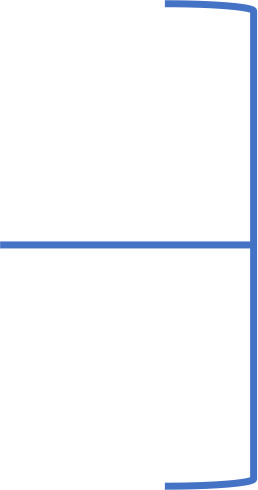	Assess the degree of subjective attention concentration, task difficulty and symptom provocation
Session 2-7	ABM & feedback on self-perception and adverse reactions		Session 4		
Session 8	ABM & summarize and feedback the whole process	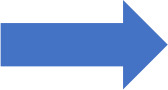	Assess changes in attentional bias and clinical symptoms		

The main indicators included accuracy, reaction time, self-reported degree of provocation, task difficulty, and attention concentration.

### 2.4 Attentional bias assessment task

**Figure 3:**
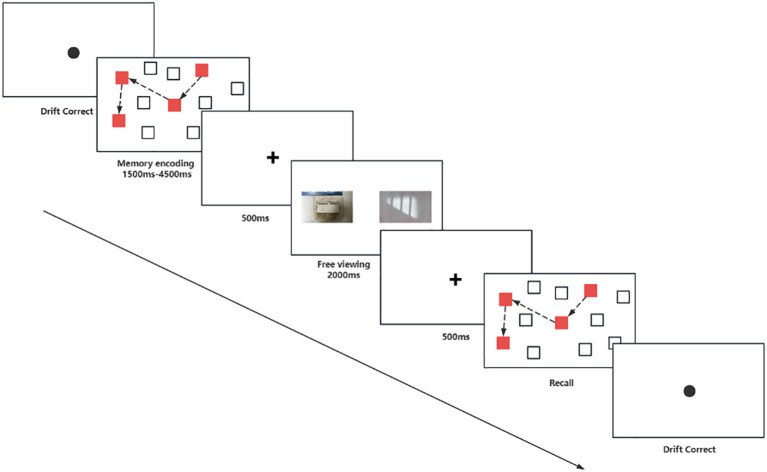
Attentional bias assessment task.

In this study, a dual-task paradigm of picture free browsing combined with visuospatial working memory task was used to further examine whether the training effects could transfer across different assessment paradigms. (**Figure 3**). Both the obsessive-compulsive pictures and neutral pictures were taken from COCSP-PS. In the eye movement task, 16 pairs of compulsive-neutral picture, 16 pairs of neutral-compulsive picture, and 16 pairs of neutral-neutral picture were randomly selected.

There were 4 exercises before the formal experiment. The formal experiment consisted of 144 trials with 48 trials in each type of picture pair, presented in random order. The main eye movement indexes included the cumulated percentage of dwell fixations (CDF), the initial fixation duration (IFD), and the direction of initial fixation (DIF). CDF was calculated as the difference in total dwell time between obsessive-compulsive and neutral pictures and was used to reflect overall attentional bias and attentional avoidance. IFD was calculated as the difference in initial fixation duration between obsessive-compulsive and neutral pictures and was used to reflect difficulty in attentional disengagement during the early and middle stages of attentional processing. DIF was defined as the proportion of trials in which the initial fixation was directed toward obsessive-compulsive pictures, reflecting attentional vigilance during early attentional processing.

### 2.5 Equipment

Eyelink1000 produced by SR Research Company was used to collect eye tracking data of left eye, with a sampling rate of 1000 Hz, 60 cm away from the screen, a screen resolution of 1280 × 1024, and a pixel of 1024 × 768 for presenting pictures.

The treatment hardware is HTC VIVE-P130, which is a VR headset, containing two 3.5-inch OLEDs, with a resolution of 1440 × 1600 in one eye and 3K (2880 × 1600) for both eyes. The locator is the HTC VIVE, and the sensor is Steam VR tracking technology 2.0.

### 2.6 Data analysis

Given the exploratory and feasibility-oriented nature of this pilot study, intervention-related outcomes were analyzed using a completer analysis. Completers were defined as participants who completed the four-week follow-up period and had both baseline and post-intervention assessments. An intention-to-treat analysis was not conducted because the very small sample size and missing post-intervention data would have required imputation based on assumptions that could not be adequately evaluated in the present study. In addition, the primary aim of this pilot study was to evaluate feasibility, acceptability, tolerability, and preliminary applicability, rather than to provide a confirmatory test of treatment efficacy. Therefore, no inferential statistical tests were conducted because of the small number of completers. To explore whether pre-post-intervention symptom changes exceeded measurement error, reliable change was assessed using the reliable change index (RCI) (35). For each clinical outcome, the standard error of difference was calculated as follows: Sdiff = SD_pre_ × √[2(1 − r)], where SD_pre_ represents the baseline standard deviation of the corresponding measure, and r represents the reliability coefficient of that measure. The RCI for each participant was then calculated as: RCI = (X_post_ − X_pre_)/Sdiff. Because lower scores indicate symptom improvement on the Y-BOCS, OCI-R, BAI, and BDI, a negative RCI indicated symptom reduction, whereas a positive RCI indicated symptom worsening. A value of |RCI| ≥ 1.96 was used as an exploratory threshold for reliable change. Although RCI values were calculated from each participant’s pre-post-intervention change score, the findings were interpreted and reported at the group level as the number and proportion of participants classified as showing reliable improvement, no reliable change, or reliable deterioration. The RCI classifications were not used as inferential evidence of treatment efficacy.

Data Viewer 4.3.1.0 and MATLAB were used to preprocess eye tracking data. RCI was calculated using R software, version 4.4.3. The clinical data, eye movement data and VR data were analyzed by descriptive statistics.

## 3 Results

### 3.1 Indicators related to VR-ABM training experience

**Figure 4:**
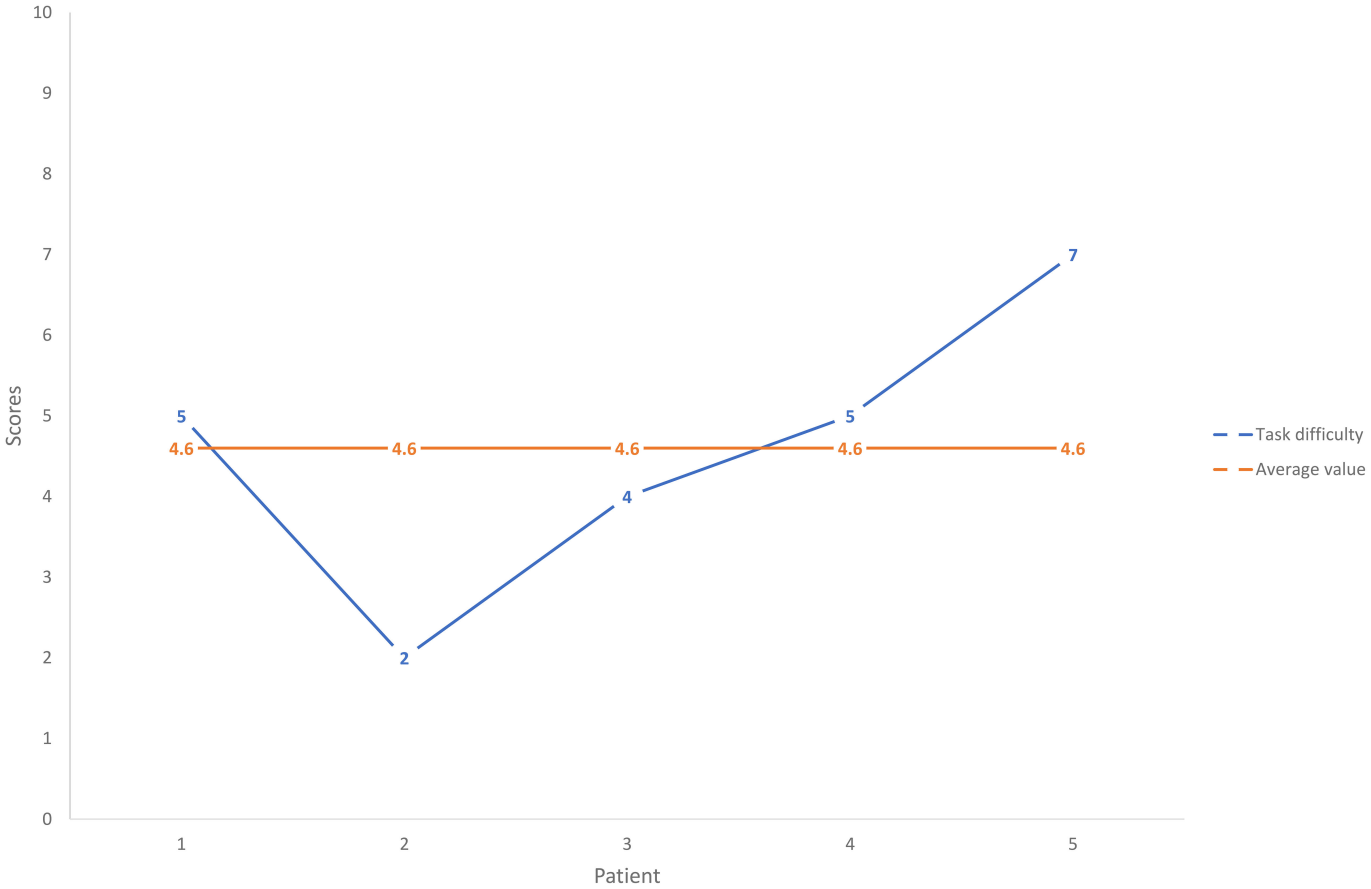
Task difficulty assessed by each patient.

Task difficulty was assessed by each patient after the first completion of ABM training (0–10 points, 0 for very simple, and 10 for very difficult), with an overall average of moderate difficulty (4.6 points) (**Figure 4**).

**Figure 5:**
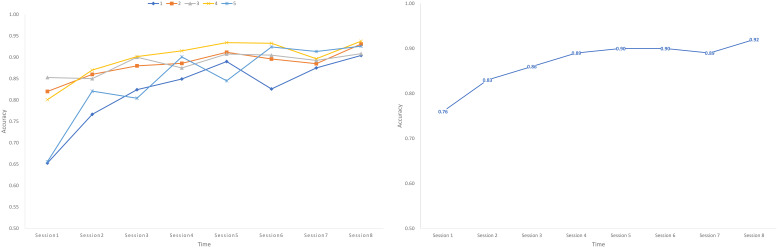
The accuracy of ABM for each patient and the average accuracy of each training session.

**Figure 6:**
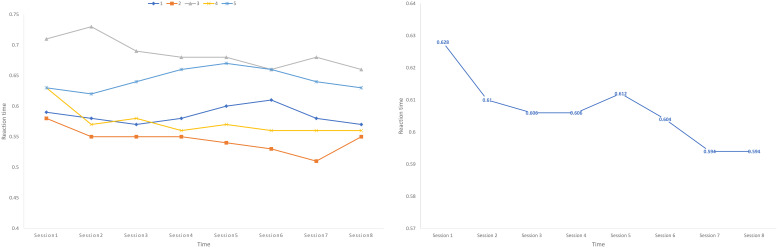
The reaction time for each patient and the average reaction time of each training session.

The accuracy of ABM training task was maintained above 50% for each patient. With the increase of training times, the average accuracy increased (change rate of 21.05%), and the average reaction time showed a decreasing trend (change rate of 5.41%) (**Figures 5**, **6**).

**Figure 7:**
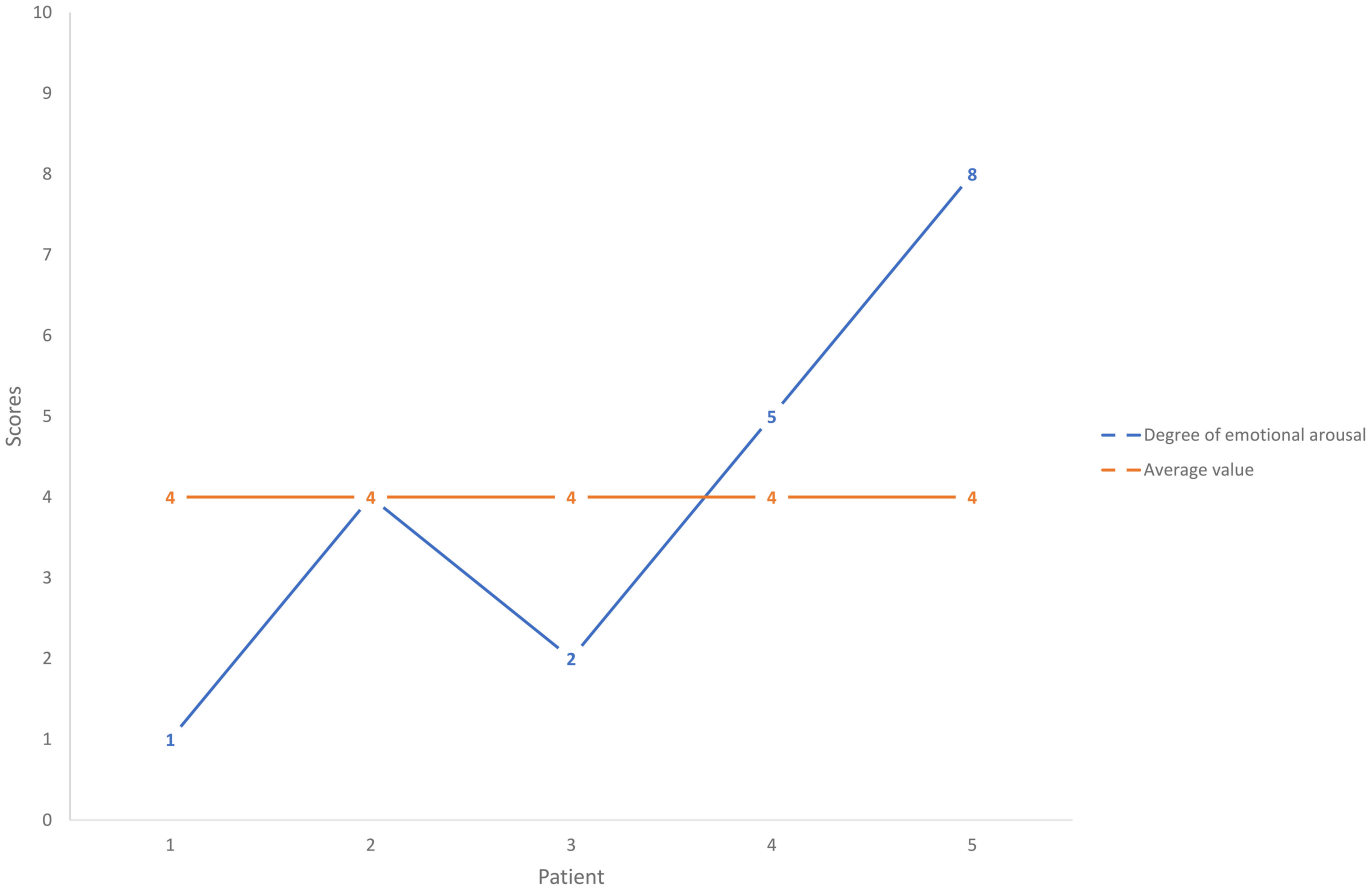
Degree of emotional arousal per patient.

After completing ABM training for the first time, patients assessed the degree to which VR scenes could provoke OCS and anxiety (0-10points, 0 being not provoked at all). The overall average degree of provocation was 4 points (**Figure 7**).

### 3.2 Objective and subjective reported indicators of attention

**Figure 8:**
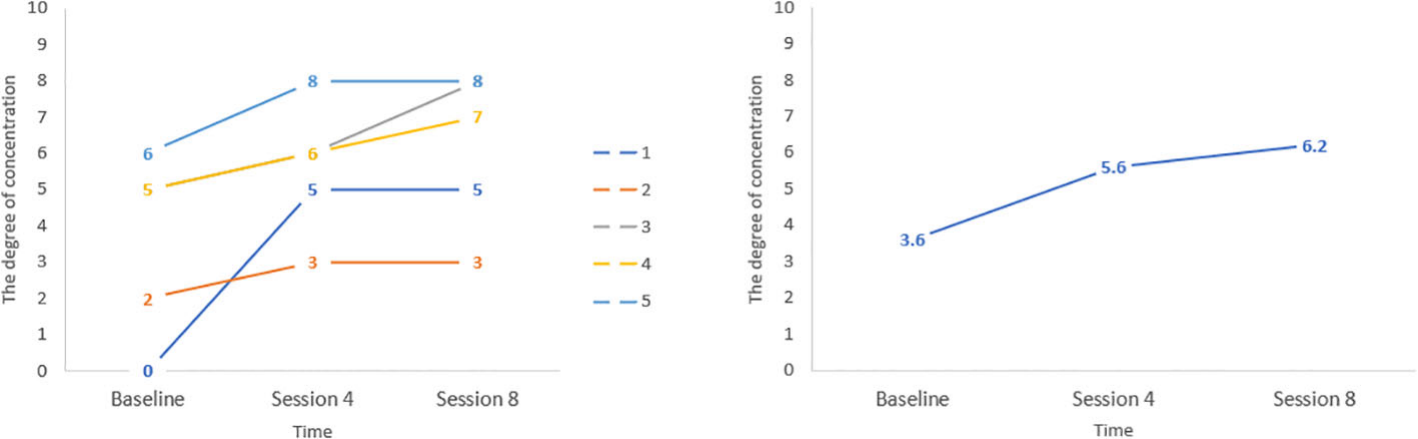
The degree of concentration of each patient and the average degree of concentration.

After the 1st, 4th and 8th training sessions, each patient was assessed for their subjective attention concentration in daily life. The degree of subjective attention concentration showed an upward trend (**Figure 8**).

Overall attentional bias and avoidance, difficulty in attentional disengagement, and early attentional vigilance were assessed using dwell fixations (CDF), the initial fixation duration (IFD), and the direction of initial fixation (DIF), respectively. After four weeks of training, three patients showed an increase in CDF, while two patients showed a decrease. For IFD, four patients decreased, and one patient increased. Two patients had a decrease in DIF, while three patients had an increase. As for TAU group, CDF and IFD decreased in three patients and increased in two patients. For DIF, three patients increased, and two patients decreased (**Table 5**).

**Table 5:** The characteristics of attentional bias at baseline and after training.

ID	Group	base			4w		
		CDF	IFD	DIF	CDF	IFD	DIF
1	ABM	-88.052	-37.417	0.604	174.617	-42.720	0.500
2	ABM	163.781	34.684	0.467	547.277	99.457	0.551
3	ABM	179.542	21.594	0.609	39.543	-8.441	0.697
4	ABM	228.677	53.033	0.593	393.292	52.862	0.500
5	ABM	417.245	44.383	0.611	204.349	38.531	0.682
6	TAU	20.750	-10.466	0.670	-82.453	32.235	0.691
7	TAU	-107.047	-130.488	0.427	-283.123	-145.145	0.429
8	TAU	259.755	-21.165	0.535	181.758	29.626	0.410
9	TAU	-742.177	-112.646	0.702	-914.137	-163.744	0.513
10	TAU	274.811	107.133	0.467	497.765	68.631	0.483

CDF, the cumulated percentage of dwell fixations; IFD, the initial fixation duration; DIF, the direction of initial fixation.

### 3.3 Subjective feedback of VR-ABM

Similarly, we sought feedback from each patient on VR-ABM training after the first, fourth, and eighth sessions, including thoughts on the task, factors affecting accuracy, more impressive scenes, suggestions for training, and side effect (**Table 6**).

**Table 6:** Patient feedback on VR-ABM training.

ID	Thoughts during the task	Factors affecting accuracy	Impressive scenes	Suggestions	Side effects
1	focused on the task	The accuracy was high when focused on the task. One mistake would lead to many mistakes.	toilet-related scenes	The olfactory experience could be more authentic.	no
2	thought of a real-life scene	The accuracy was high in cleaner scenes. News scenes with sound were less accurate.	toilet with vomit	It may be more effective to adjust perspective, add sound, and make the scenes dirtier.	no
3	guessed where the letters would appear and focus on the task	The accuracy was higher when the letters were in the middle. One mistake would lead to many mistakes.	stained floor, toilet-related scenes	If the scenes are dynamic, the effect may be better.	no
4	focused on the task and accuracy	The accuracy of news scenes with sound was low.	toilet-related scenes	The scenes were not dirty enough.	no
5	thought of a real-life scene	The accuracy was low when the mind was wandering and in the news scene with sound.	infectious disease related news scenes, toilet-related scenes	It would be better to have more scenes and elements.	no

### 3.4 Changes of clinical symptom-related indicators

In ABM group, the average reduction rates of Y-BOCS and OCI-R were 24.40% and 6.78%, respectively. For the degree of anxiety, the BAI score decreased in four patients, and increased in one patient after training. The average score-reducing rate of BAI was 24.99%. The degree of depression decreased in one patient, increased in two patients, and scores remained unchanged in two patients. And the average score reduction rate of BDI was -5.36%. In TAU group, there were four patients with reduced Y-BOCS and OCI-R scores, with mean reduction rates of 9.86% and -29.86%. BAI scores decreased in two patients and BDI scores decreased in two patients. The average score-reducing rate of BAI and BDI was 0% and -65.50%, respectively (**Table 7**).

**Table 7:** Clinical symptoms and their reduction rate before and after 4 weeks.

ID	Group	Base				4w				ΔY-BOCS	ΔOCI-R	ΔBAI	ΔBDI
		Y-BOCS	OCI-R	BAI	BDI	Y-BOCS	OCI-R	BAI	BDI				
1	ABM	21	25	17	9	15	32	11	9	0.286	-0.280	0.353	0
2	ABM	18	32	21	3	13	30	22	3	0.278	0.063	-0.048	0
3	ABM	18	22	19	13	15	21	14	17	0.167	0.045	0.263	-0.308
4	ABM	15	27	3	22	14	14	2	17	0.067	0.481	0.333	0.227
5	ABM	26	34	23	32	15	33	15	38	0.423	0.029	0.348	-0.188
	ABM M(SD)	19.60 (4.16)	28.00 (4.95)	16.60 (7.93)	15.80 (11.39)	14.40 (0.89)	26.00 (8.22)	12.80 (7.26)	16.80 (13.24)	0.244 (0.135)	0.068 (0.271)	0.250 (0.170)	-0.054 (0.204)
6	TAU	18	38	40	6	16	38	40	6	0.111	0	0	0
7	TAU	21	38	21	12	21	34	14	13	0	0.105	0.333	-0.083
8	TAU	21	23	2	3	16	19	3	1	0.238	0.174	-0.500	0.667
9	TAU	19	49	12	14	15	46	10	12	0.211	0.061	0.167	0.143
10	TAU	15	12	0	1	16	34	0	5	-0.067	-1.833	0	-4.000
	TAU M(SD)	18.80 (2.49)	32.00 (14.51)	15.00 (16.31)	7.20 (5.63)	16.80 (2.39)	34.20 (9.81)	13.40 (15.87)	7.40 (5.03)	0.099 (0.132)	-0.299 (0.860)	0 (0.312)	-0.655 (1.893)

Y-BOCS, The Yale-Brown Obsessive-Compulsive Scale; OCI-R, Obsessive-compulsive Inventory-Revised; BAI, Beck Anxiety Inventory; BDI, Beck Depression Inventory; Δ, the reduction rate.

**Figure 9:**
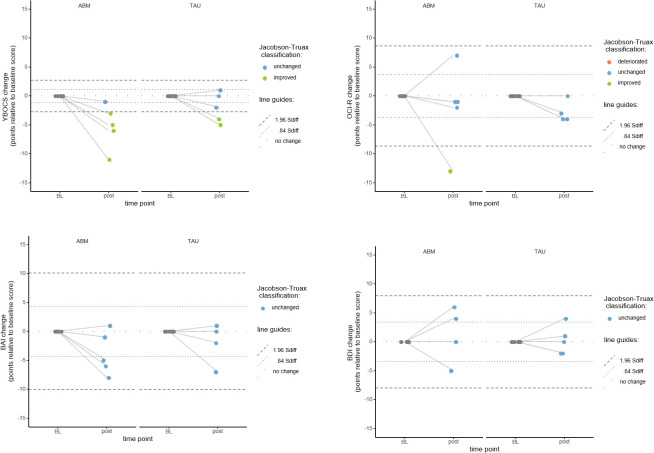
Jacobson-Truax (criterion A) classification of individual clinical symptoms change across time.

**Figure 9** showed the RCI (Jacobson-Truax classification) of Y-BOCS, OCI-R, BAI, and BDI for each patient. There were four patients whose Y-BOCS scores improvement was beyond the range of measurement error in ABM group, which indicated that the symptom improvement was reliable. In TAU group, there were two patients improved in Y-BOCS. As for OCI-R scores, only one patient improved in ABM group. There was no reliable improvement in BAI and BDI scores in either group.

## 4 Discussion

To our knowledge, this study is the first to integrate contamination-related virtual scenes with a visual search task to develop a novel VR-ABM training program for individuals with OCD. The present findings should be interpreted as preliminary and exploratory because the completer analysis included only five participants per group and the outcomes were summarized using descriptive statistics. Nevertheless, the findings provide a valid basis for cautious preliminary inferences regarding feasibility and potential clinical applicability. First, all completers underwent the same predefined VR-ABM protocol, which ensured procedural consistency across participants. Second, the outcome evaluation included both subjective ratings and objective behavioral indices recorded within the VE, thereby reducing reliance on self-report alone. Third, the two groups were balanced in size, and the observed descriptive patterns were generally consistent across several feasibility-related indicators. Specifically, the VR-ABM program elicited a moderate degree of symptom provocation (mean provocation score = 4) and task difficulty (mean difficulty score = 4.6). Furthermore, all patients demonstrated the ability to complete the task with an accuracy rate exceeding 50%, suggesting its potential feasibility and applicability. Following the four-week training period, participants showed a decrease in response times and an increase in accuracy, with no reported adverse effects, providing preliminary support to its suitability. Moreover, changes in both subjective and objective measures of attentional processes were observed, indicating that the program may contribute to enhance attentional allocation in individuals with OCD. However, findings regarding changes in attentional bias were inconsistent, necessitating further investigation. While this study was primarily designed to establish and validate the feasibility of VR-ABM as an intervention, we also noted a reduction in Y-BOCS symptom scores when VR-ABM was administered in conjunction with pharmacotherapy, with a clinically relevant mean reduction rate of 24.40%. Given the small completer sample and descriptive nature of the analyses, these results should not be interpreted as confirmatory evidence of efficacy. Rather, they provide preliminary feasibility evidence and a methodological foundation for future studies with larger samples, control conditions, and inferential statistical analyses.

This study empirically selected scenes (public restroom, hospital, garbage station) or elements (stains, blood stains, vomit, urine, infectious disease-related news broadcasts, etc.) that were threatening to most OCD patients with contamination as the main symptom dimension. As in previous studies, these VR scenes can effectively provoke OCS and anxiety in patients (36–39). Although the average level of symptom provocation in the training scene of this study was moderate, there was considerable heterogeneity in symptom provocation among patients. First, this is related to the introduction used during the training in this study. The therapist informed patients that they needed to respond more quickly and accurately to judgment tasks. For instance, patients with IDs 1 and 3 reported lower levels of symptom provocation, indicating that their thoughts during the task were “focused on the task”. In contrast, patients with IDs 2 and 5 reported higher levels of symptom provocation; both participants noted that they were thought of relevant real-life scenarios during the task. Overall, based on patients’ self-reports and clinical researchers’ observations, the level of symptom provocation remained within a relatively safe range (1-8). However, higher levels of provocation might increase the difficulty of the training and decrease patients’ compliance. This suggests that the symptom provocation level of scenes in our VR-ABM training can be managed through introduction that encourage patients to concentrate more on the task, thereby avoiding excessive symptom provocation. To further enhance the scenes, based on patient feedback, it may be beneficial to consider increasing relevant stimuli in visual, auditory, olfactory, and interactive dimensions.

We investigated patients’ feedback on task experience during training. Before starting the training, patients reported that they were unable to concentrate in daily life. They were more likely to notice symptom-related stimuli than others and found it difficult to shift their attention. The results of eye tracking tasks also reflected that patient had more fixation time and frequency on OCD-related pictures than on neutral pictures. After the first training session, three patients reported that they would associate real-life scenes related to contamination when they saw stimuli when completing tasks. But patients were aware that they were completing the task and can quickly shift their attention from the symptom-related stimuli back to the target to continue with the task. After treatment, four patients had less difficulty in attentional disengagement, two patients had less attentional vigilance, and the degree of attentional avoidance decreased in two patients. The characteristics of attentional bias in the ABM group were improved compared with the TAU group. At the same time, all patients reported an increase in concentration during tasks and daily life as training progressed. Robbins (40) posits that individuals with OCD exhibit an imbalance in habit and goal-directed system, potentially linked to impaired top-down control. ABM may, therefore, improve patients’ top-down attentional control. A review examining alterations in brain network activity during ABM (10) revealed activation of the dorsal attention network (DAN) – a network implicated in top-down attention–and the executive control network (ECN), which is involved in cognitive control and emotional self-management (41–43). Consequently, ABM may enhance the patients’ ability of active allocation of attention resources, promote a shift of attention away from negative stimuli, and improve the ability to focus on task-relevant stimuli. This echoes the plasticity of attentional control mentioned in the background. This change in attentional control was also initially observed in our study.

In this study, all patients had been steadily taking SSRIs for more than one month. Within the ABM group, baseline symptom severity for one participant was mild, for three was moderate, and for one was severe. During the training period, four patients reported emotional stability, three patients reported a reduction in time and frequency of cleaning compared to baseline, and one reported less fear of pollutants in her life after training. After training, the average score reduction rate of Y-BOCS in five patients was 24.40%, and the severity of symptoms was reduced to mild. According to RCI, four patients improved effectively. The reduction rates of BAI were 24.99%. In TAU group, the severity of symptoms in one patient was mild, four were moderate in baseline. After 4 weeks, there were two patients improved in Y-BOCS scores. The improvement of symptoms in ABM group was better than that in TAU group. This was consistent with previous meta-analysis studies showing small to moderate effect sizes for ABM on symptom and emotional relief (44–46). The results of seven existing VR-ABM studies have also shown a reduction in symptoms and corresponding emotions in participants after training. But there is no consensus on the effectiveness of ABM training. Rouel (47) and Habedank (8) performed a dot-probe task using OCD-related and neutral picture pairs, completing a single and six sessions of ABM training, respectively. The results showed a reduction in attentional bias, but there was no significant difference in symptom reduction compared with TAU group. This may be related to the different type of task and stimulus materials.

In addition, we found that Y-BOCS and BAI scores decreased after VR-ABM training, whereas no significant reductions were observed in OCI-R or BDI scores. This discrepancy may be related to differences in the constructs and assessment formats captured by these measures. The Y-BOCS is a clinician-rated measure that primarily captures the overall severity of core OCS. As such, it may be more sensitive to changes in the global severity of OCS following the intervention. In contrast, the OCI-R is a self-report measure that emphasizes symptom dimensions across different OCD domains. Because our sample was composed mainly of patients with contamination symptoms, the OCI-R total score may not have been sufficiently sensitive to detect changes in the predominant symptom domain. In addition, prior studies have shown that self-report and clinician-rated OCD measures demonstrate only moderate agreement and may differ in symptom coverage (48). Therefore, short-term improvement detected by clinician-rated severity measures may not be immediately reflected in a broader self-report index. Similarly, the lack of a significant reduction in BDI scores may suggest that depressive symptoms were not the most immediate treatment target of VR-ABM. This interpretation is broadly consistent with previous findings (49) indicating that ABM has shown clearer effects on anxiety-related outcomes, whereas findings for depression have been more mixed. Given the small sample size and the brief 4-week intervention period, these discrepancies across outcome measures should be interpreted cautiously and further examined in studies with larger samples and longer follow-up periods.

Although comorbidity is common in OCD (50, 51), we excluded major psychiatric comorbidities in this pilot study to reduce heterogeneity and improve interpretability. Severe disorders may significantly affect cognitive processing, attentional bias, symptom presentation, and treatment response (52), thereby making it more difficult to isolate the specific feasibility and preliminary effects of VR-ABM in OCD. Importantly, participants were not entirely free of emotional symptoms. Some participants still had anxiety and depressive symptoms, although these did not meet criteria for an independent primary diagnosis. Therefore, the sample retained some clinical characteristics commonly seen in OCD while avoiding severe comorbid conditions that might confound the evaluation of VR-ABM. In addition, the aim of this study was to preliminarily develop and validate a novel attention bias modification paradigm. As a proof-of-concept study, it was necessary to first establish its feasibility and safety in a relatively homogeneous OCD sample before extending it to more complex clinical populations. Future studies with larger and more representative samples should include OCD patients with more complex comorbid presentations.

Moreover, final group allocation was adjusted according to patient preference after initial randomization and before treatment initiation. Although this may have improved feasibility and retention in this pilot study (53), it may also have reduced the benefits of randomization and introduced selection bias (54). Participants choosing VR-ABM may have differed from those in TAU in motivation, treatment expectations, or willingness to engage with the intervention. Therefore, the between-group findings should be interpreted as preliminary.

## 5 Limitations

First, this study is an exploratory pilot study with a small sample size, which can only preliminarily explore the feasibility of VR-ABM but cannot prove its effectiveness. Secondly, only the most common scenes related to compulsive washing are selected. The number of scenes is small, and there is no personalized allocation for patients. Third, the intervention period lasted only 4 weeks, which may not have been sufficient to produce stable or clinically meaningful changes. In addition, because no follow-up assessments were conducted, the durability of the intervention effects over time could not be determined. Moreover, OCS were assessed using scale-based outcome measures only at baseline and at the four-week follow-up, which may not fully capture fluctuations in symptom severity or the multidimensional nature of OCS. The strict inclusion criteria, as well as the secondary randomization based on patients’ preferences, may limit the interpretability of the results and reduce the generalizability of the findings. Future studies should include larger samples, adopt more rigorous randomized controlled designs, incorporate long-term follow-up assessments, and use more comprehensive and repeated assessments, potentially combining clinician-rated, self-report, behavioral, and ecological measures, to further evaluate the effectiveness and durability of the intervention. In addition, expanding the scene library and developing individualized training tailored to patients’ specific symptom profiles may help optimize the intervention effect.

## 6 Conclusion

This study implemented a VR-ABM program, derived from the principles of COCPS, which incorporated 3D-modeled images and visual search tasks. Five OCD patients completed eight training sessions. Participants’ subjective feedback indicated that the program was of appropriate difficulty and could provoke OCS and anxiety moderately. Throughout training, participants self-reported a reduction in their clinical symptoms and improved concentration, both during the sessions and in daily activities. No side effects were reported during the training. Overall, participant experience was positive, with suggestions made to increase the program’s immersion and realism. While this study provides initial support for the feasibility and acceptability of the VR-ABM program, its limitations (including small sample size) necessitate further research. Future studies should prioritize larger, randomized controlled trials to definitively evaluate the program’s efficacy and to guide further refinements of the intervention and optimize its clinical application.

## Data Availability

The original contributions presented in the study are included in the article/supplementary material. Further inquiries can be directed to the corresponding author.
